# On the analysis of movement smoothness

**DOI:** 10.1186/s12984-015-0090-9

**Published:** 2015-12-09

**Authors:** Sivakumar Balasubramanian, Alejandro Melendez-Calderon, Agnes Roby-Brami, Etienne Burdet

**Affiliations:** Department of Bioengineering, Christian Medical College, Bagayam, Vellore, 632002 Tamil Nadu India; Department of Physical Medicine and Rehabilitation, Northwestern University, Chicago, 60611 IL USA; Hocoma AG, Volketswil, 8604 Switzerland; ISIR, UPMC, CNRS UMR 7222, Agathe team INSERM U1150, Paris, 75005 France; Department of Bioengineering, Imperial College of Science, Technology and Medicine, South Kensington campus, London, SW7 2AZ UK

**Keywords:** Smoothness, Sensorimotor assessment, Discrete movements, Rhythmic movements

## Abstract

Quantitative measures of smoothness play an important role in the assessment of sensorimotor impairment and motor learning. Traditionally, movement smoothness has been computed mainly for discrete movements, in particular arm, reaching and circle drawing, using kinematic data. There are currently very few studies investigating smoothness of rhythmic movements, and there is no systematic way of analysing the smoothness of such movements. There is also very little work on the smoothness of other movement related variables such as force, impedance etc. In this context, this paper presents the first step towards a unified framework for the analysis of smoothness of arbitrary movements and using various data. It starts with a systematic definition of movement smoothness and the different factors that influence smoothness, followed by a review of existing methods for quantifying the smoothness of discrete movements. A method is then introduced to analyse the smoothness of rhythmic movements by generalising the techniques developed for discrete movements. We finally propose recommendations for analysing smoothness of any general sensorimotor behaviour.

**Electronic supplementary material:**

The online version of this article (doi:10.1186/s12984-015-0090-9) contains supplementary material, which is available to authorized users.

## Introduction

*This work was developed as part of the project “State of the Art Robot-Supported assessments (STARS)” in the frame of the COST Action TD1006 “European Network on Robotics for NeuroRehabilitation”* [1]*. The goal of STARS is to give neurorehabilitation clinical practitioners and scientists recommendations for the development, implementation, and administration of different indices of robotic assessments, grounded on scientific evidence.*

A movement is perceived to be smooth, when it happens in a continual fashion without any interruptions. Smooth movements are a characteristic feature of healthy and well-trained motor behaviour [2]. Movement smoothness increases with neural development [3], motor learning [4] and motor recovery after a stroke [5]. Movement smoothness has been identified as an important marker of motor recovery in patients with stroke [5, 6], which correlates with standard scales of motor impairments such as the Fugl-Meyer assessment [7]. Smooth movements are believed to be the result of effort minimisation [8, 9] – an important characteristic of motor learning [10]. Smoothness may also be related to other fundamental determinants of sensorimotor control such as spatio-temporal coordination.

Although smoothness can provide valuable information about sensorimotor control and be used for patient’s assessment during neurorehabilitation, there is little consensus on the best method to quantify smoothness. For instance, at least 8 different measures have been used in various studies [4], which makes it difficult to compare the results from these different studies. The choice of factors to assess sensorimotor control (such as smoothness) is generally based on some observed invariants of healthy/trained sensorimotor behaviour. For any such chosen factor, the conception of a *measure* for its evaluation is often guided by a model of healthy behaviour. In the case of movement smoothness, nearly all existing measures are motivated by the stereotypical smooth kinematics of discrete arm movements of healthy subjects, i.e. the single peaked bell-shaped speed profile (left trace of Additional file 1: Figure S1) of point-to-point reaching [11–13].

Movements that do not follow this kinematic pattern (e.g. point-to-point movements with several speed peaks shown in the right trace in Additional file 1: Figure S1) are perceived as being less smooth. The family of jerk measures [4, 14] are all based on the minimum jerk model [12], as minimal jerk trajectories correspond well to the reaching movements of healthy subjects. The correlation between a speed profile and the corresponding minimum jerk speed profile has also been used as a smoothness measure [15]. The number of peaks measure [16] relies on the fact that the speed profile of smooth movements are single peaked, while unsmooth movements have higher number of speed peaks. More recently, we have introduced the spectral arc length as a measure of smoothness [4], which relies on changes in the Fourier spectrum of movements to quantify smoothness.

On the other hand, it must be noted that the available smoothness measures have primarily focused on discrete arm movements, such as point-to-point reaching and circle drawing [17]. A few studies have also investigated the smoothness of the kinematics of other body segments, such as the head [18], jaw [19], elbow [20, 21], forearm rotation [22], wrist [23], lower-extremity [24–27] etc. Additionally, some studies have also investigated the smoothness of rhythmic movements [28], including walking [24–26, 29], back-and-forth elbow flexion/extension [21], and rhythmic object manipulation [30].

In the gait literature, the harmonic ratio has been used as a measure of smoothness [24–26, 29]. The harmonic ratio (HR) is defined as the ratio of the sum of the magnitudes of the even harmonics to that of the odd harmonics of the trunk acceleration over a single stride [29]. The smoothness of the entire gait data is analysed by segmenting the data into individual strides. HR exploits the inherent periodicity of gait and is primarily a measure of gait symmetry of the two legs [29]. Although symmetry may be an important component of how one would visually judge the overall smoothness of a person’s gait, it is a very different factor from that of smoothness. For instance, one may walk with perfectly symmetric but unsmooth (very intermittent) gait pattern. Therefore HR does not appear to be an appropriate measure of smoothness.

In the case of the upper-extremity, Nasseroleslami et al. [30] estimated the smoothness of kinematic and force data from a rhythmic object manipulation task. They converted their (almost periodic) rhythmic movements [28] into single averaged cycles, to which the spectral arc length measure [4] was applied. Ao et. al [21] used a similar approach for studying the smoothness of rhythmic elbow flexion/extension movements of stroke subjects. Here also, entire rhythmic movements were segmented into individual flexion and extension movements, to which the dimensionsless jerk measure was applied [4]. The common approach in these studies analysing rhythmic movements is to segment the long rhythmic movement into individual discrete segments to which existing measures are applied. However, currently there is no systematic investigation on the best method to analyse smoothness of the different types of rhythmic movements [28]. It is important to point out that Hogan and Sternard [28] proposed the use of mean squared jerk as a measure of smoothness of entire discrete and rhythmic movements. However, mean squared jerk is not a valid measure of smoothness [4], which was noticed by the same authors in a later paper [14].

In general, smoothness has only been discussed in the context of movement kinematics, with a few exceptions such as [30] which have looked at the smoothness of force profiles. Smoothness in the context of sensorimotor control is merely a measure of intermittency, which can be applied to contexts other than movement kinematics such as isometric force and torque. However, we are not aware of any other studies than [30] investigating the smoothness of force/torque profiles of isometric tasks, or the impedance profiles of impedance modulation tasks.

The present paper yields a first step towards a general approach for analysing movement smoothness, with the goal to enable a systematic analysis of any movement type. The paper first provides a definition of movement smoothness, and the factors that affect a movement’s smoothness. Following this, a brief review of the relevant smoothness measures in the current literature is provided, along with a modified version of the spectral arc length from [4] to address the original measure’s sensitivity to temporal scaling of movement data. This is followed by the presentation of a general approach to estimating movement smoothness, which can be used to analyse any movement type. Finally, the paper presents a set of recommendations for the analysis of smoothness, which we hope will help in the standardisation of this type of movement analysis.

## What is movement smoothness?

*Movement smoothness* is a quality related to the continuality or non-intermittency of a movement, independent of its amplitude and duration. Intermittency in this context refers to movements that alternately decelerate and accelerate, and more intermittency corresponds to un-smooth movements. Movement intermittency is typically observed as dips in a movement’s speed profile or finite non-zero periods of zero speed (i.e. movement arrest) during an ongoing movement. A dip in a speed profile is a point where the second derivative of position goes to zero, and it highlights a period of deceleration followed by acceleration, which is a mark of movement intermittency. On the other hand, a movement arrest period represents an extreme form of intermittency where all derivatives of position go to zero for a finite non-zero duration. The longer the arrest period, the more intermittent the behaviour. It must be noted that this does not mean that a constant state of rest represents an extreme form of intermittency. Movement arrest periods are “unwanted” periods of rest occurring in the context of an ongoing change or movement, and thus are major contributors to movement intermittency.

Movement intermittency can be observed in upper-extremity movements of infants below 6 months [3], in slow movements [20, 31], movements requiring accuracy [32], and in patients with neurological conditions such as stroke [5], multiple sclerosis [33], Parkinson’s disease [34, 35] etc. Reaching arm movements of healthy adult subjects have almost straight line trajectories with bell-shaped speed profiles [11]. However, the same task performed by stroke subjects can be highly intermittent, with the hand accelerating and decelerating several times on its way to the target [5] as shown in the simulated example of Additional file 1: Figure S1.

An important point to note here is that intermittencies can occur in a movement due to two distinct set of factors:

1. **Control level/ability of a performer on a specific task:** The current and most important use of smoothness in movement analysis is its use to infer the control ability of a subject. This factor leads to intermittencies caused by poor motor planning/execution due to neurological impairments, lack of familiarity with the task or the environment, and/or injury to the musculoskeletal system. For instance a healthy subject’s movements in a novel task or novel dynamic environment can be intermittent due to poor task/environment familiarity. Intermittent movements of stroke patients are often the result of poor motor control caused by the neurological injury. In patients with stroke, the control ability of a subject would have a direct relationship to the level of injury to the neural tissue. The lack of smoothness in stroke affected movements result from poor temporal blending of submovements [36]. However, the neurophysiological basis of this remains unclear. The segmentation of movements could be a consequence of the disruption of upper-limb coordination, interruptions due to the triggering of spasticity, pathological movement synergies or directly to an impairment of the temporal organisation of the movement. Changes in the cortico-spinal tract excitability following stroke [37] could also be one of the contributors to the observed impairment of movement behaviour. Although the direct relationship between cortico-spinal tract excitability and smoothness is not clear, smoothness could serve as a simple global measure of the cortico-spinal tract integrity.
2. **Nature of the task or task constraints:** The nature of a task can lead to intermittencies that are not the result of poor control but are necessitated by its constraints or requirements. For instance, point-to-point reaching movements through a via point performed by healthy subjects can exhibit sharp dips in the speed profile [12]. Task constraints determine the minimum amount of intermittency (or the maximum smoothness) in a movement. Therefore **smoothness is strongly task-dependent**, and **one cannot simply compare the smoothness of two completely different tasks**, e.g. point-to-point reaching versus circle drawing. This task-dependence must be considered when analysing and comparing smoothness of any general task type such as discrete and rhythmic tasks. Note that this is never explicitly discussed in the current literature as existing studies usually focus on the analysis of a single type of task.

Given these two distinct factors that can contribute to movement intermittency, smoothness could be used as a criterion for differentiating between (a) control abilities of different subjects when the task is controlled; and (b) different types of tasks when skill is controlled. The former type of analysis is what one typically encounters in motor learning and neurorehabilitation literature. The latter analysis was discussed by Hogan and Sternard in [28] where they propose mean squared jerk as a criterion for differentiating between discrete and rhythmic movements. However, this can be done only if the two movements were performed by the same subject or by two subjects that are equally skilled.

## What is a good smoothness measure?

A *smoothness measureλ*_*s*_ is a function

(1) $$ \text{Smoothness} = \lambda_{s}\left(M_{m} \left| T\right.\right)   $$

that characterises a movement’s smoothness, where *M*_*m*_ represents some measured information about the given movement, e.g. kinematics; and *T* represents the task, e.g. point-to-point reaching, target tracking, or some spatio-temporal constraints. The task-dependence of smoothness is indicated by *T* in Eq. 1.

A good estimate of movement smoothness requires a measure that is *valid*, *sensitive*, *reliable* and *practical*. *Validity* is the most important property, which refers to whether or not a given measure estimates the factor it is intended to measure [38]. Sensitivity and reliability deal with how well a given measure is able to quantify a movement related factor. *Sensitivity* is essential for a measure to resolve real but small differences in a factor. A measure must have good sensitivity, particularly in the physiological range that covers the entire spectrum of healthy and un-healthy movements. *Reliability* is directly related to the robustness of the measure to measurement noise^1^, i.e. the amount of variability introduced in the measure by a given level of measurement noise. *Practicality* refers to issues related to the practical implementation of the measure, which needs to consider factors such as computational complexity of the measure (e.g. how does the computational time increase with increase in data size).

For movement smoothness, a valid measure must be independent of the movement amplitude and duration, i.e. *dimensionless*. Smoothness is measured through the amount of movement intermittency, which is directly related to the movement’s temporal organization or coordination. Thus, a valid smoothness measure must change monotonically to changes in movement intermittency. For example, if a given movement can be thought of as a superposition of discrete submovements, then an increase in the number of submovements or the interval between two successive submovements must result in decreased movement smoothness. This can be understood from Additional file 1: Figure S1, where the healthy movement has one submovement while the stroke affected movement has three submovements with a finite temporal gap between two successive submovements. A simulated scenario was used to investigate the validity of different existing smooth measures in [4], where simulated movements were generated by systematically varying the number of submovements and the inter-submovement interval.

## Smoothness measures - *status quo*

The current neurorehabilitation and motor control literature reports several different measures for estimating smoothness of a given discrete movement [4, 14–16, 36, 39, 40]; these are are listed in Table 1 along with the details of their different properties. The most common measures are the jerk-based measures [14], most of which are not valid measures of smoothness. The dimensionless jerk (DLJ) and the log dimensionless jerk (LDLJ), defined below, are the only valid jerk-based measures of movement smoothness [4]:

(2) $$\begin{array}{@{}rcl@{}}  \text{DLJ} & \triangleq & -\frac{\left(t_{2}-t_{1}\right)^{5}}{v_{peak}^{2}}\int_{t_{1}}^{t_{2}} \left|\frac{d^{2}v(t)}{dt^{2}}\right|^{2}\,dt \\ \text{LDLJ} & \triangleq & -\ln\left|\text{DLJ}\right| \end{array} $$

**Table 1:** Technical properties of different existing smoothness measures

Measure	Validity			Sensitivity	Reliability	Practicality
	D	M1	M2		Measurement noise	
Root mean square jerk	×	-	-	-	-	✓
Normalized mean absolute jerk	×	-	-	-	-	✓
Dimensionless jerk	✓	✓	✓	×	×	✓
Log dimensionless jerk	✓	✓	✓	✓	×	✓
No. of peaks	✓	✓	✓	×	×	✓
Speed arc length	✓	✓	✓	✓	×	✓
Spectral arc length						
(SAL introduced in [4])	×	✓	✓	✓	✓	✓
Spectral arc length						
(SPARC introduced in this paper)	✓	✓	✓	✓	✓	✓

D - Dimensionless; M1 - Monotonic response to changes in submovement interval; M2 - Monotonic response to changes in number of sub-movements. (× means the measure does not satisfy this property, $\checkmark $ indicates that it does satisfy the property. − indicates that information about this property is not available)

where *v*(*t*) is the movement speed, *t* is time, *t*_1_,*t*_2_ are the start and end times of the movement, and $v_{\textit {peak}} \triangleq \max _{t \in \left [t_{1}, t_{2}\right ]}v(t)$ is the peak speed. DLJ lacks sensitivity in the physiological range [4], which the LDLJ addresses through the natural log function. However, both DLJ and LDLJ are very sensitive to measurement noise and have poor reliability [4].

The other most commonly used smoothness measure is the number of peaks (NP) measure that counts the number of maxima in a given speed profile *v*(*t*):

(3) $$  \text{NP} \triangleq -\left|\left\{v(t)\,, \,\,\frac{dv(t)}{dt}=0 \,\, \text{and} \,\, \frac{d^{2}v(t)}{dt^{2}}<0\right\}\right|  $$

where $\left |\cdot \right |$ represents the cardinality of a set. NP is a simple measure, but lacks sensitivity and robustness [4].

The spectral arc length measure (SAL) introduced in [4] uses a novel approach to estimate smoothness. It estimates smoothness by calculating the arc length of the Fourier magnitude spectrum within the frequency range 0 to 20 Hz of a given speed profile *v*(*t*):

(4) $${} \text{SAL} \triangleq -\int_{0}^{\omega_{c}}\!\left[\!\!\left(\!\frac{1}{\omega_{c}}\!\right)^{2} \,+\, \left(\!\frac{d\hat{V}(\omega)}{d\omega}\!\right)^{2}\!\right]^{\frac{1}{2}}\!d\omega; \,\,\, \hat{V}(\omega) = \frac{V(\omega)}{V(0)},  $$

where *V*(*ω*) is the Fourier magnitude spectrum *v*(*t*), $\hat {V}(\omega)$ is the normalized magnitude spectrum, normalized with respect to the DC magnitude *V*(0), and *ω*_*c*_ is fixed to be 40*π* (corresponding to 20 Hz).

In this paper, we introduce a slightly modified version of the original SAL, which we call SPARC for **SP**ectral **ARC** length, by setting

(5) $$ \omega_{c} \triangleq \min\left\{\omega_{c}^{max}, \min\left\{\omega\,\,\,,\hat{V}(r)<\overline{V} \,\,\, \forall \,\,\, r >\omega \right\} \right\}   $$

The SPARC extends SAL in that *ω*_*c*_ is adaptively selected based on a given threshold $\overline {V}$ and is upper-bound by $\omega _{c}^{max}$. In contrast to SAL, SPARC is independent of temporal movement scaling, and retains the good sensitivity and reliability of the SAL. A detailed explanation of the SPARC measure and the rationale for its development is given in Appendix A of the Additional file 2. The entire analysis presented in the paper was carried out using iPython [41]. The entire analysis can be found online at https://github.com/siva82kb/SPARC.

## A general measure of smoothness - looking beyond discrete movements

A general measure of movement smoothness is one that can be applied to any movement type, e.g. discrete or rhythmic; and on any movement-related data, e.g. kinematics, forces, impedance etc. Let us consider a general measure of movement smoothness *λ*_*s*_ where *T* in Eq. (1) is any type of sensorimotor task. Let **x**(*t*) represent the measured information associated with a motor action *M*_*m*_ that is generated in response to the given task *T*:

$$ \mathbf{x}(t) = \left[x_{1}(t), x_{2}(t), x_{3}(t), \cdots, x_{N}(t)\right]^{T} $$

where **x**(*t*) is the measured movement related variable, *x*_*i*_(*t*) is the *i*^*th*^ component of **x**(*t*), and *t* represents time. The information contained in **x**(*t*) can be movement kinematics, force or even mechanical impedance, in either task or joint space, depending on the task and the sensing modality used for measuring the motor behaviour. For example, movement kinematics could be simple spatial or joint space location measured through a motion tracking system, or linear accelerations and angular rates measured using an inertial measurement unit. In the case of isometric tasks, it would contain force or torque in the task or joint space.

How does one analyse the smoothness of any type of task? The simplest method would be to apply an existing smoothness measure on **x**(*t*) independent of the task type. However, this approach does not work, as we demonstrate through a simple example involving a rhythmic movement. Let us consider a simulated experiment where three subjects (two experts and one novice) were instructed to move back-and-forth 10 times between two spatial targets P and Q. The subjects were asked to move at a comfortable self-selected speed, and rest at the targets for a short duration. The movements *M*_*a*_, *M*_*b*_ and *M*_*c*_ made by the three subjects are shown in Additional file 3: Figure S2. The smoothness estimates obtained by simply applying the SPARC and LDLJ measures to the entire movement data are also shown in these plots in Additional file 3: Figure S2.

By visual inspection of the position data in Additional file 3: Figure S2, one immediately concludes that the movement *M*_*b*_ is less smooth than *M*_*a*_ and *M*_*c*_, as *M*_*b*_ is more intermittent during some individual movements between P and Q. The smoothness estimates shown in Additional file 3: Figure S2 agree with this conclusion, as *M*_*b*_ is less smooth than *M*_*a*_ and *M*_*c*_ using both SPARC and LDLJ. The movement time (MT) and dwell-time (DT) of *M*_*a*_ and *M*_*b*_ are exactly the same. Thus, differences between their smoothness estimates is due to differences in intermittency of the individual movement components (movements between P and Q) of *M*_*a*_ and *M*_*b*_.

Now, by applying the same logic, can we say anything about the smoothness of *M*_*a*_ and *M*_*c*_? Is one smoother than the other, or are they both equally smooth? Using the same argument as before, we would conclude that *M*_*a*_ and *M*_*c*_ are equally smooth, because in both *M*_*a*_ and *M*_*c*_, the movements between the targets P and Q appear similar (except in *M*_*c*_ the movements are a little faster, the dwell-time is shorter). The results in Additional file 3: Figure S2, however, do not agree with our intuition in this case; smoothness of *M*_*a*_ and *M*_*c*_ are close but not equal. This difference is due to the sensitivity of SPARC and LDLJ to the temporal organisation of the individual movement components in the entire rhythmic movement (i.e. the relative values of MT and DT). When the SPARC and LDLJ are applied on an entire rhythmic movement, it is seen as one long discrete movement, with the temporal organisation of the individual movement components appearing as intermittencies, which affects the overall smoothness. This implies that changes in the number of components will also result in a change of the smoothness estimate. For example, consider another movement *M*_*d*_ (plot not shown) which has the same MT and DT as *M*_*a*_ but has 20 back and forth movements between P and Q instead of 10 in *M*_*a*_. Here, the smoothness of *M*_*a*_ is higher than that of *M*_*d*_ (value shown in the bottom plot in Additional file 3: Figure S2), when in fact the smoothness of *M*_*d*_ is no different from that of *M*_*a*_ and *M*_*c*_. Therefore, the conclusion is that the **smoothness measures cannot be used on an entire rhythmic movement to estimate smoothness**.

In light of the foregoing discussion, our intuitive judgement of a rhythmic movement’s smoothness appears to be based on the smoothness of the distinct individual components in the movement. It also appears to be independent of the number of such components and their temporal organisation in the overall rhythmic movement. Additionally, we have illustrated how using SPARC or LDLJ to evaluate smoothness on the entire movement does not match this intuitive judgement. Thus, generalising from the above discussion, **we can define smoothness of a rhythmic movement as a function of the smoothness of its individual, distinct (non-overlapping) movement components**. This way of defining the smoothness of a rhythmic movement agrees with our intuition that the properties of the parts (i.e. the distinct components) must influence the properties of the whole (i.e. the entire rhythmic movement). This implies that existing measures can be used to evaluate smoothness of the individual movement components, and also the overall smoothness of the movement.

Consider a movement represented by **x**(*t*), $t \in \left [t_{s}, t_{e}\right ]$, where *t*_*s*_ and *t*_*e*_ are the start and end times of the movement, respectively. This movement **x**(*t*) can be represented as a concatenation of a set of individual distinct (non-overlapping) movement components^2^**x**^*i*^(*t*):

(6) $$ \begin{aligned} \mathbf{x}(t) &= \sum_{i=1}^{N}\mathbf{x}^{i}(t) \,;\,\,\, \mathbf{x}^{i}(t) = \mathbf{x}(t)\Pi_{i}(t) \,; \\ \Pi_{i}(t) &= \left\{\begin{array}{cc} 1 & \quad t_{i} \leq t < t_{i+1} \\ 0 & \text{otherwise} \end{array}\right. \end{aligned}   $$

where the *i*^*th*^ distinct movement component **x**^*i*^(*t*) is the product of the **x**(*t*) and the rectangular window *Π*_*i*_(*t*); *N* is the number of distinct components in **x**(*t*), and *t*_*i*_ is the starting time of the *i*^*th*^ movement component, where *t*_1_=*t*_*s*_ and *t*_*N*+1_=*t*_*e*_, and $t_{i} < t_{i+1}, \,\forall i \in \left \{1,2,3,\cdots,N\right \}$. We note that the representation in Eq. (6) is not unique, and one can choose the value of *N* and the *t*_*i*_s arbitrarily. For movements *M*_*a*_ and *M*_*b*_ in Additional file 3: Figure S2, one possible representation in terms of Eq. (6) would consist of *N*=20 and $t_{i}=1.25\left (i-1\right)$. This particular choice of values for *N* and *t*_*i*_ for *M*_*a*_ and *M*_*b*_ would segment out the individual discrete movements between the targets P and Q, along with postures at one of the targets. One can come up with a similar model for *M*_*c*_ with the parameters *N*=20 and $t_{i}=0.7\left (i-1\right)$. The representation in Eq. (6) is a form of windowing procedure to segment the long movement into meaningful components for which the smoothness can be estimated individually; we call this the **event-based segmentation** procedure. With this procedure one can estimate the overall smoothness of a given movement by first estimating the smoothness of its individual components, and then combining the individual estimates to obtain a single number representing the “overall” smoothness *Λ* of the movement **x**(*t*):

(7) $${} \text{Overall Smoothness}\left(\Lambda\right) = f\left(\left[\lambda_{1}, \lambda_{2}, \lambda_{3}, \cdots, \lambda_{N}\right]\right)   $$

where *f*(·) is a mathematical function that combines the smoothness estimates of the individual movement components in **x**(*t*), *λ*_*i*_ is the smoothness estimate of the *i*^*th*^ movement component obtained using the selected measure e.g. SPARC. The choice of function for *f*(·) is crucial, and this must be a function that ensures that the overall smoothness *Λ* satisfies some of the following properties:

1. If all the segmented components of a movement correspond to the same type of task (e.g. point-to-point reaching like in Additional file 3: Figure S2) and have the same smoothness $\left (\lambda _{1} = \lambda _{2} = \lambda _{3} = \cdots = \lambda _{N} \equiv \lambda \right)$, then the smoothness of the overall movement must be equal to *λ*.
2. *f*(·) must be independent of the ordering of the individual movement components, i.e. permuting the values of the *λ*_*i*_s must not affect the overall smoothness *Λ*.
3. The overall smoothness value *Λ* of a movement must be no greater than the smoothness of the most smooth component, and no less than that of the least smooth component, i.e.
  $$ \min_{i\in\left\{1,2\cdots N\right\}} \left\{\lambda_{i}\right\} \leq \Lambda \leq \max_{i\in\left\{1,2\cdots N\right\}} \left\{\lambda_{i}\right\} $$
  Note that property 1 is implied by this property.

Based on these properties, a good function for estimating the overall smoothness of a given movement from its components is the *weighted average function*:

(8) $$ \Lambda = \frac{\sum_{i=1}^{N}w_{i}\lambda_{i}}{\sum_{i=1}^{N}w_{i}}; \,\,\, \sum_{i=1}^{N}w_{i} \neq 0   $$

where *w*_*i*_≥0 is the weight given to the smoothness of the *i*^*th*^ movement component *λ*_*i*_, and the denominator in Eq. 8 is a normalising factor ensuring that property 3 is satisfied.

A nice feature of the weighted average scheme is that it can be used to summarise the smoothness of an entire movement or just specific parts of a movement. For example, consider the movement *M*_*a*_ in Additional file 3: Figure S2. Let us decompose this movement using Eq. (6) with parameters *N*=20, and *t*_*i*_=1.25(*i*−1). Now, to estimate the overall movement smoothness one could choose $w_{i} = 1 \,\, \forall i \in \left \{1,2,\cdots N\right \}$. But if we only wanted the overall estimate of all movements from P to Q in *M*_*a*_ (i.e. excluding movement from Q to P), then one could choose *w*_*i*_=1 when *i* is odd and 0 otherwise. Moreover, the event-based segmentation scheme for smoothness estimation of movements also allows one to track the smoothness over the course of movement, on an event-by-event basis.

An important point to note about the general smoothness measure of Eqs. (6) and (8) is that the results of smoothness analysis strongly depend on the movement segmentation step, the details of which are controlled by the parameters *N* and *t*_*i*_ in Eq. (6). Although, technically they could be chosen arbitrarily, **one must ensure that the choice of parameters*N* and*t***_***i***_** yields meaningful segmentation of the movement data**. That is, the movement components should be clearly identified as specific events in the overall movement, or they could be specific events of interest for the purpose of an analysis. In any analysis, one must first choose the events of interest, which will guide the choice of segmentation parameters and the subsequent smoothness estimation. For example, a meaningful segmentation for movements in Additional file 3: Figure S2 would be to segment out individual movements between P and Q. In general, the segmentation step can be guided by metadata obtained from, (a) knowledge about the task that was being performed (e.g. task type, target locations, via-points, movement reversals etc.); (b) annotations^3^ of the movement data recorded during data collection; and (c) also from the actual movement data^4^.

Some form of movement segmentation appears to be the most preferred method, in the current literature, for analysing smoothness of rhythmic movements. Nasseroleslami et al. [30] had segmented (almost periodic) rhythmic movements into individual cycles, which were averaged before estimating the smoothness of averaged cycles. Even though here the approach was similar to the proposed general smoothness measure, the smoothness estimation process was different from that of Eq. (8). On the other hand, Ao et al. [21] and the gait studies [24, 25, 27, 29] can be seen as instances of the general approach described in this section. Ao et al. segmented (almost periodic) rhythmic movements based on the desired trajectory that was displayed to their subjects to follow [21]. While, the gait studies analysing smoothness using the HR, segmented data based on heel strikes detected from foot switch data collected during gait [25, 29]. However, these studies did not evaluate an “overall” estimate of the entire movement, unlike the proposed general measure.

## How to measure smoothness?

### Systems for measurement

The systems for measurement depend on the tasks and movement variables of interest. Most existing studies have been on discrete movements, using robotic devices [6, 23, 36], motion capture systems [21] or inertial measurement units [42] for measuring kinematics. Table 2 provides a non-exhaustive list of possible sensing systems that can be used. Notwithstanding the modality, sensors for measuring motor actions should not introduce excessive measurement noise. Noise will distort the smoothness estimate, with lower signal-to-noise ratio (SNR) resulting in more distortion. With the LDLJ, even signals with SNR = 100 can severely distort smoothness estimates, while the SPARC appears to be relatively immune up to SNR = 10, and possibly even lower. Please refer to reliability analysis in Appendix B in the Additional file 2 for more details.

**Table 2:** Systems for measuring the different variables of interest for different types of tasks

Task	Variables of interest	Measurement system
Movement task	Position	Robotic devices or camera-based motion capture system to measure position in either task space or joint space. Wearable potentiometers for measuring joint position.
	Acceleration, angular rate	Inertial measurement units.
Isometric task	Force/Torque	Robotic devices for both task space and joint space measurements. Multi-axis load cells for measuring force and torque in different directions.
Movement or isometric task	Impedance	Robotic devices for measuring task or joint space impedance through appropriate perturbation methods. EMG recording of opposing muscle pairs to measure joint stiffness.

### Protocols for assessment

Sensorimotor assessments provide a clinician/therapist an idea about a subject’s overall sensorimotor capability, providing a quantitative basis for tracking a subject’s recovery with time and therapy. Ideally, tasks performed in an assessment should be good global indicators of sensorimotor ability, i.e. performance on these tasks generalizes to a wide range of real-world activities. Movement smoothness is an important marker of recovery [4, 7]. Planar point-to-point reaching [36], and circle drawing [17] tasks have been the most popular choices for assessing movement smoothness in stroke. Improvements in smoothness made in tasks trained during therapy also generalize to movements not explicitly trained as part of therapy [17], indicating that smoothness can provide a general measure of overall control ability. Thus, when designing an assessment protocol one need only use a small set of tasks to assess smoothness. Based on the current literature, point-to-point reaching movements are good candidates for assessing movement smoothness of the upper-extremity. No such suggestions can be made for the lower-extremity given the dearth of information regarding the smoothness of lower-extremity movements.

### What smoothness level is normal?

Sensorimotor neurorehabilitation aims at improving movement capability, ideally, targeting a level of performance before the injury. Thus, any sensorimotor assessment requires the range of scores expected from a healthy individual undergoing the assessment. For standard clinical scales, the normative range is defined as part of the scale. Similarly, sensor-based quantitative assessments must define the corresponding normative ranges for the different tasks and measures used in the assessment. For movement smoothness, the normative scores depend on the task being performed. Point-to-point reaching tasks are the only well-studied tasks in the literature, which would have scores around –1.6 for the SPARC and around –6 for the LDLJ [4]. More studies are required to obtain estimates of the normative ranges for the different tasks.

## Recommendations for measurement

As a final step in the discussion of movement smoothness analysis, we would like to list recommendations for researchers and clinicians interested in analysing movement smoothness.

### Smoothness is task-dependent

The task-dependence of smoothness is an important point to take into consideration. As discussed in an earlier section, movement smoothness is equally affected by both changes in a subject’s control ability and the task constraints. Thus, smoothness analysis to assess a subject’s control ability must account for the task variable to remove its confounding effect. This applies to the smoothness analysis of any movement type, and one must exercise caution particularly in the case of rhythmic movements, where an artificial movement segmentation step is introduced. In the case of discrete movements, one can only compare tasks of similar nature. This obviously leads to a question about the similarity of tasks. Tasks are usually described in qualitative terms, e.g. point-to-point reaching, reaching through a via-point etc. In quantitative terms, tasks could be described as spatiotemporal constraints, which a motor behaviour needs to conform to, in order to achieve a goal. In the context of smoothness, tasks can be considered similar if they can be achieved with the same level of intermittency. For example, all simple point-to-point reaching tasks are similar in nature as they exhibit bell-shaped speed profiles. By the same argument a simple point-to-point reaching task cannot be compared to that of task with a via point, because a via point requires a more intermittent movement than the one without a via point. Similarly, point-to-point tasks with varying number of via points are not similar.

Likewise, in the case of rhythmic movements, comparisons can be made only between rhythmic movements where the underlying tasks are similar. For rhythmic movements in Additional file 3: Figure S2, the individual movements between the targets P and Q, and the overall movement smoothness (estimated using Eqs. 6 and 8) can be compared to each other, but these movements cannot be compared to other rhythmic movements like repetitive figure drawing or walking, where the task constraints are quite different to those in Additional file 3: Figure S2.

### Avoid estimating smoothness of unconstrained movements

Given the strong task-dependence of smoothness, one cannot meaningfully interpret the smoothness of unconstrained movements – movements that are not goal-directed, and are completely exploratory in nature. In such cases, without the knowledge of what was attempted one cannot know whether the observed movement intermittency is planned or unplanned. Thus, nothing can be said about the relative contributions of the task and the subject’s skill to the overall smoothness of these movements.

### All measurements are not the same

Our objective in this paper was to formulate a general measure of movement smoothness that can be applied to any movement type, using any movement variable (e.g. kinematics, kinetics, impedance etc.). The generalisation of the smoothness measure to any movement type was presented in an earlier section. However, so far we have not explicitly talked about the different types of movement variables or movement-related data.

Analysis of smoothness is most likely to be used with movement kinematic data either in the task or the joint space. However, it is possible to investigate smoothness of any sensorimotor behaviour using e.g. measurements of force, torque or even impedance associated with the behaviour. Most of the current literature on smoothness analysis has been on movement kinematics, where derivatives of position (e.g. speed, jerk etc.) are used to estimate smoothness. The derivative operation is used for two reasons:

1. smoothness is relevant only in the context of a change. It does not make sense to talk about the smoothness of a fixed posture. A derivative will help get rid of any movement component that does not change over time.
2. derivatives of position highlight movement intermittencies.

It is not immediately clear how one would use either the SPARC or LDLJ, if the measured variable was either acceleration, angular rate, force or impedance. Given that these variables are of different nature, it is important to treat each of them appropriately to estimate smoothness using these variables. Table 3 provides some suggestions for processing the different movement related variables before estimating smoothness. It must be noted that many of these suggestions are not based on any experimental work, and must be verified for their appropriateness in future studies involving these variables.

**Table 3:** Proposed methods to process different types of measured movement related variables before applying the SPARC or LDLJ measures

Measured movement variable x (t)	Proposed processing for SPARC and LDLJ		Rationale
Movement kinematics - position (in either task or joint space) through motion capture system or other position sensors	SPARC:	Use speed, $\lVert \frac {d\mathbf {x}(t)}{dt} \rVert _{2}$	Speed highlights intermittencies, and does not amplify noise as much as the other higher order derivatives.
	LDLJ:	Use jerk magnitude, $\lVert \frac {d^{3}\mathbf {x}(t)}{dt^{3}} \rVert _{2}$	This is by definition.
Movement kinematics - acceleration measured by an accelerometer	SPARC:	Use gravity subtracted absolute magnitude of acceleration, $\left |\lVert \mathbf {x}(t) \rVert _{2} - g\right |$	Accelerometers also pick up gravity, and this must be removed to apply the SPARC, otherwise this would lead to a large DC component in the spectrum, which will dominate the other spectral components. This proposed method is based on our unpublished prior work on estimating smoothness from accelerometers. It must be noted that here the SPARC is used on signals from the acceleration space, and not the velocity space, as was done with position information.
	LDLJ:	Use magnitude of jerk, $\lVert \frac {d\mathbf {x}(t)}{dt} \rVert _{2}$	This is by definition. Simply derive the jerk from the accelerometer data.
Force, Torque or impedance	SPARC:	Use the magnitude of first derivative of force/toque, $\lVert \frac {d\mathbf {x}(t)}{dt} \rVert _{2}$	The proposed method for SPARC and LDLJ are based on the treating these variables like position variables. This suggestion is purely based on intuition, and must be verified through future experiments.
	LDLJ:	Use the magnitude of third derivative of force/torque, $\lVert \frac {d^{3}\mathbf {x}(t)}{dt^{3}} \rVert _{2}$	

### More noise results in less smoothness for any movement

In general, smoothness measures make use of a derivative operation to quantify the amount of intermittency in a movement; this is the primary reason for their sensitivity to noise. To minimise the effect of noise one should use a reliable smoothness measure more robust to measurement noise. The SPARC measure is more robust to measurement noise than the LDLJ for different signal-to-noise ratios (SNR). Figure S4 and Figure S5 in the Additional file 2 demonstrate this, and the details of the analysis are provided in Appendix B in the Additional file 2. If any post-processing is carried out to suppress high frequency noise (such as using a lowpass filter), one must ensure that the entire data used in the analysis are put through the same noise reduction process. For example, the same movement when filtered differently can result in different smoothness estimates.

### Same noise results in less smoothness for slow movements

Smoothness measures are affected by noise, and changes in SNR in movement data can affect smoothness estimates differently (Figure S4 and Figure S5 in Additional file 2); the bias and variability in a smoothness estimate increases with decrease in SNR. The SNR of a given movement data is determined by the amount of signal power and the amount of noise power. In any given movement recording set-up, the measurement noise can be assumed to have a constant power. Under such a condition, slower movements would have a lower SNR than faster movements, which could mean that the smoothness of slower movements might be underestimated compared to that of the faster movements, even when both the (uncorrupted) movements’ speed profiles have the same shape, except for the scaling of the amplitude and duration. This was found to be the case using simulated data (Figure S6 of the Additional file 2. Details of this analysis are provided in Appendix C of the Additional file 2). Figure S6 in the Additional file 2 shows that the SPARC measure is less sensitive to changes in SNR than the LDLJ. This is another important aspect to be aware of when analysing smoothness of movements with very different speeds. Thus, one must always use a reliable measure such as SPARC for estimating smoothness.

### Use SPARC over LDLJ

The SPARC has a better reliability than the LDLJ in the face of ubiquitous measurement noise present in all data. The LDLJ is highly sensitive to noise, and even lowpass filtering does not fully address this problem (refer to Appendix B in the Additional file 2). In light of these serious problems with the LDLJ, **we recommend the use of SPARC over LDLJ**.

## Endnotes

^1^ Measurement noise is the cumulative effect of noise in the measurement system (sensors and instrumentation), changes in assessment protocol, and also other unknown or unaccounted sources.

^2^ Note that such a model is used purely for the purpose of smoothness analysis, and it does not make any assumptions about the organising principles governing the observed movement.

^3^ Annotation could be movement metadata recorded during the data collection process. For example, in motor control experiments the computer program recording movement data and presenting audio-visual feedback during the experiment, would need to make decisions on when a subject has reached a target, or when he/she has stopped moving etc. This sort of information could be later used to segment the data for analysis.

^4^ Let us consider the example of a figure drawing task where a subject was asked to draw a large symbol of infinity on a planar surface using his/her arm, repeatedly *N* times. Here, the subject could have started from any point on the infinity symbol and drawn the shape repeatedly. This is an example of an almost periodic movement [28]. To segment this data into individual cycles representing a complete drawing of the symbol, one could define the points of segmentation as the times where the actual movement states (e.g. position, velocity, acceleration etc.) almost repeat themselves. In this case the segmentation is entirely based on the actual movement data.

## Additional files

Additional file 1
**Figure S1.** Smooth and unsmooth reaching movements. Simulated speed profiles of healthy (normal) and stroke (abnormal) reaching movements. The stroke affected speed profile is more intermittent than the healthy speed profile. (SVG 15 kb)

Additional file 2
**Appendix.** This additional file includes the details of the SPARC measure development and the analysis of its properties. (PDF 474 kb)

Additional file 3
**Figure S2.** Smoothness analysis of simulated rhythmic movements. Simulated rhythmic task consisting of self-paced back and forth reaching movements between two targets P and Q, performed by three subjects (**a**) *M*_*a*_ movement made by expert 1 (movement time (MT) = 1 sec and dwell-time (DT) = 0.25 sec), (**b**) *M*_*b*_ movement by novice (MT = 1 sec and DT = 0.25 sec), and (**c**) *M*_*c*_ movement by expert 2 (MT = 0.6sec and DT = 0.1sec). The smoothness of all these movements were estimated through SPARC and LDLJ using the entire movement data. The parameters $\overline {V}=0.05$ and $\omega _{c}^{max}=20\pi $ were used for the SPARC. (SVG 75 kb)
