# Supplementary material for: On the analysis of movement smoothness
Source: J Neuroeng Rehabil. 2015 Dec 9;12:112. doi: 10.1186/s12984-015-0090-9 (PMC4674971; doi:10.1186/s12984-015-0090-9)
Supplement: Additional file 2 — Appendix. This additional file includes the details of the SPARC measure development and the analysis of its properties. (PDF 474 kb) [file 12984_2015_90_MOESM2_ESM.pdf]

Appendix A

Drawback of the SAL

The SAL measure of movement smoothness was proposed in [1]. For a given movement speed profile $v(t)$, it is defined as the following,

$$\text{SAL} \triangleq -\int_0^{\omega_c} \left[\left(\frac{1}{\omega_c} \right)^2 + \left(\frac{d\hat{V}(\omega)}{d\omega} \right)^2 \right]^{\frac{1}{2}} d\omega; \;\; \hat{V}(\omega) = \frac{V(\omega)}{V(0)} \tag{1}$$

where, $V(\omega)$ is the Fourier magnitude spectrum of $v(t)$, $\hat{V}(\omega)$ is the normalized magnitude spectrum, normalized with respect to the DC value of the spectrum $V(0)$, and ω_c is fixed to be 40π (corresponding to 20Hz).

A drawback of this measure in Eq.(1) is the use of a fixed cut-off frequency ω_c, which causes the measure to be affected by temporal scaling (compression or dilation) of $v(t)$. This is demonstrated in Fig. 1. Fig. 1(A) displays Gaussian speed profiles of different durations, which are all temporally scaled versions of each other. The corresponding Fourier magnitude spectra of the different speed profiles is shown in Fig. 1(B). As the duration of the speed profile is changed from 0.1s to 2.0s through temporal scaling (dilation), the corresponding Fourier magnitude spectrum is compressed along the frequency axis. The use of a fixed cut-off frequency of $\omega_c = 40\pi$ (corresponding to 20 Hz), the arc length of the magnitude spectrum is different for these different speed profiles, as shown

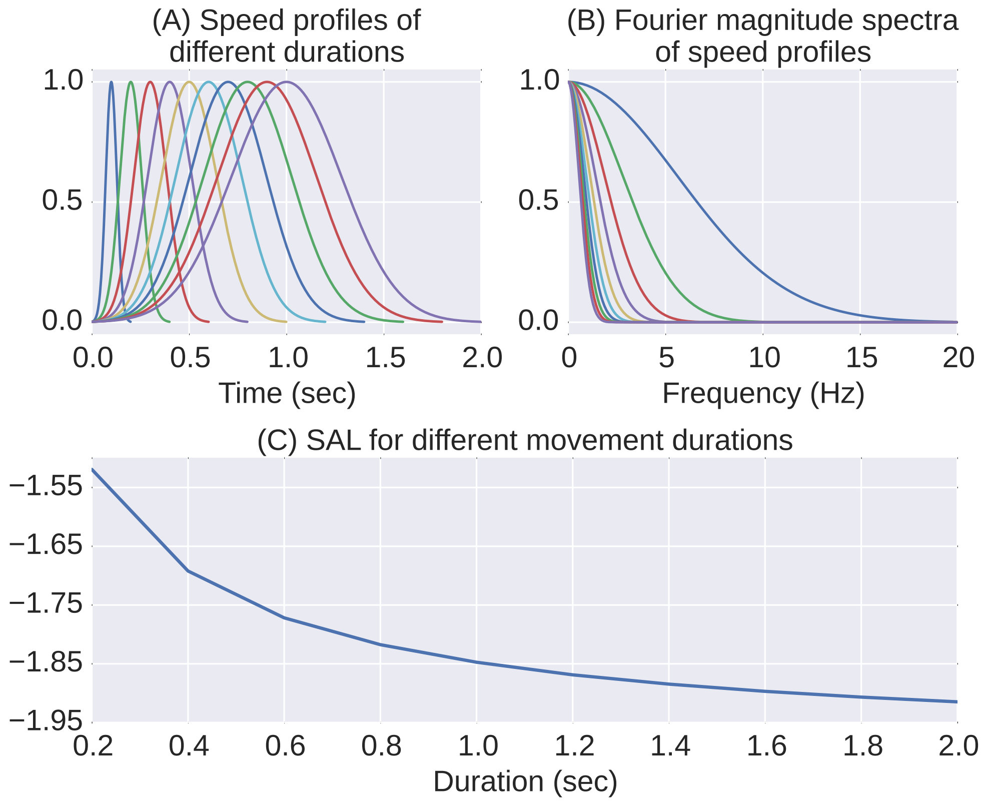

Figure 1: **Effect of temporal scaling of a Gaussian speed profile on the spectral arc length (SAL) measure defined in [1].**

in Fig. 1(C). The smoothness as measured by the SAL reduces by about 0.4, as the duration of the speed profiles increases from 0.1s to 2.0s. Smoothness values are not supposed to change with temporal scaling, as smoothness is independent of the movement amplitude and duration.

SPARC: A modified SPectral ARC length

The sensitivity of the SAL to movement temporal scaling is caused by the use of a fixed cut-off frequency ω_c to evaluate the integral in Eq. 1. A natural solution to address this problem, (i.e., to make the measure dimensionless) is to use an adaptive cut-off frequency determined from the magnitude spectrum. A simple approach is to use a threshold $\overline{V}$ on the normalized magnitude spectrum $\hat{V}(\omega)$ and choose ω_c using the following rule,

$$\omega_c \triangleq \min\left\{\omega\,,\ \hat{V}(r) < \overline{V}\ \forall\ r > \omega\right\} \tag{2}$$

This is demonstrated in Fig. 2(A), which shows the magnitude spectra of two Gaussian speed profiles of different durations. By choosing the threshold $\overline{V}$, we can determine two different cut-off frequencies (ω_{c1} and ω_{c2}) for these two movements. Calculating the smoothness of these two movements using Eq. 1 using their corresponding cut-off frequencies[1] will yield the same value for their smoothness, thus addressing the problem with the SAL.

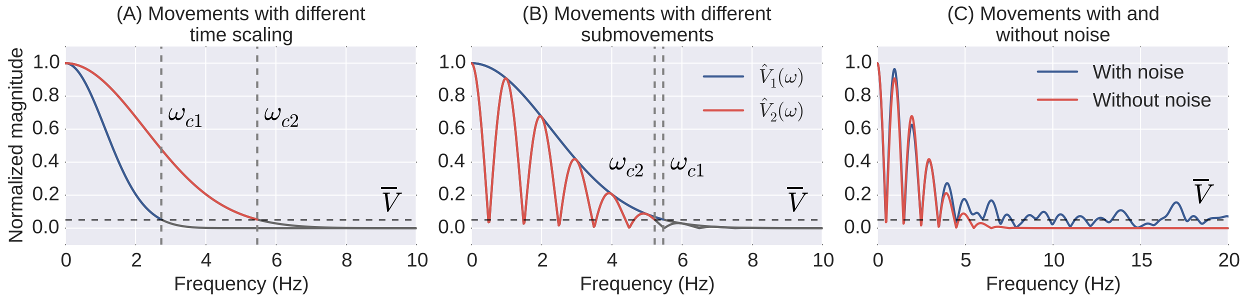

Figure 2: **Effect of the amplitude threshold $\overline{V}$ on the cut-off frequency ω_c used for estimating the SPARC. ω_c is negatively correlated to $\overline{V}$; (A) Demonstrates that adaptive selection of ω_c can address the problem with temporal scaling of movements; (B) Demonstrates that a low value for $\overline{V}$ is essential for capturing all the details in the magnitude spectrum reflecting the amount of intermittency in a movement; and (C) Demonstrates that a high value for $\overline{V}$ is required to make the SPARC measure more robust to measurement noise.**

How can one choose $\overline{V}$? The choice of value for $\overline{V}$ affects two different properties of the measure: (a) the measure's sensitivity, i.e. its responsiveness to changes in movement intermittency; and (b) its reliability, i.e. its robustness to noise. These two aspects are illustrated in Fig. 2(B) and Fig. 2(C), respectively. Fig. 2(B) shows two spectra $\hat{V}_1(\omega)$ and $\hat{V}_2(\omega)$; $\hat{V}_1(\omega)$ corresponds to a movement with a single Gaussian submovement, while $\hat{V}_2(\omega)$ corresponds to a movement with two Gaussian submovements with non-zero inter-submovement interval. The plot also displays the cut-off frequencies (dashed vertical lines) corresponding to these two movements, along with the portions of their spectra above these cut-off frequencies (grey traces). It is clear from $\hat{V}_2(\omega)$, that choosing a high value for $\overline{V}$ will result in a lower cut-off frequency ω_{c2}, and thus ignore details in the spectrum beyond ω_{c2}, which contains information about the movement's intermittency. This discarding of spectral details results in a corresponding loss of sensitivity in the measure, which can be minimized by keeping the value of $\overline{V}$ as low as possible.

[1] Using ω_{c1} and ω_{c2} for the two corresponding movements, instead of fixed cut-off $\omega_c = 40\pi$ for both movements.

On the other hand, a low value for $\overline{V}$ will make the measure very sensitive to noise in the movement data, as shown in Fig. 2(C). When a speed profile is affected by noise, the magnitude spectrum may never go to zero (blue trace in Fig. 2(C)), and thus might result in a very large value for the cut-off frequency[2]. In Fig. 2(C), the spectrum of the noisy movement signal (blue trace) never goes below the threshold $\overline{V}$, when compared to the corresponding ideal movement (red trace). Thus, to prevent choosing a very high cut-off frequency because of noise, the selection of the cut-off frequency must be upper-bound to an appropriate value ω_c^{max}:

$$\omega_c \triangleq \min\left\{\omega_c^{max}, \min\left\{\omega\,,\ \hat{V}(r) < \overline{V}\ \forall\ r > \omega\right\}\right\} \tag{3}$$

where ω_c^{max} is the upper-bound for the cut-off frequency and its value is chosen to include the frequency band of both normal and abnormal movements. This value can be set to 40π corresponding to 20Hz.

Thus, the SPARC measure of movement smoothness is defined as the following,

$$\begin{aligned}\text{SPARC} &= -\int_0^{\omega_c}\left[\left(\frac{1}{\omega_c}\right)^2 + \left(\frac{d\hat{V}(\omega)}{d\omega}\right)^2\right]^{\frac{1}{2}} d\omega\,;\quad \hat{V}(\omega) = \frac{V(\omega)}{V(0)}\\ \omega_c &= \min\left\{\omega_c^{max}, \min\left\{\omega\,,\ \hat{V}(r) < \overline{V}\ \forall\ r > \omega\right\}\right\}\end{aligned} \tag{4}$$

The SPARC measure requires the choice of two parameters, $\overline{V}$ and ω_c^{max}. A recommended choice for these two parameters are $\overline{V} = 0.05$ and $\omega_c^{max} = 20\pi$.

SPARC Algorithm

The algorithm for calculating the SPARC measure is as follows:

1. Segment speed profile to select the part of the data corresponding to the movement $\{v[n]\,,\ n \in \{0,\ldots,N-1\}\}$.

2. Compute Fourier magnitude spectrum. Compute the K-point fast Fourier tranform (FFT) magnitude spectrum of the speed profile by padding $(K-N)$ zeros to the speed profile.

$$V[k] = |FFT(v_{zp}[n])|,\ k \in \{0,\ldots,K-1\}$$

$$v_{zp}[n] = \begin{cases} v[n] & 0 \le n \le N-1 \\ 0 & N \le n \le K-1 \end{cases}$$

$$K = 2^{\mathrm{roundup}(\log_2 \mathrm{N})+4}$$

3. Normalize Fourier magnitude spectrum with respect to its DC value

$$\hat{V}[k] \triangleq \frac{V[k]}{V[0]}$$

It must be noted that a speed signal always has a non-zero DC component $V[0]$, so the normalization will not run into a division-by-zero problem.

4. Choose the cut-off frequency. Choose the cut-off frequency using the chosen threshold $\overline{V}$. This is the discrete-time and discrete-frequency equivalent of Eq. 3.

$$K_c \triangleq \min\left\{K_c^{max}, \min_K\left\{K \,\middle|\, \hat{V}[r] < \overline{V}\ \forall r > K\right\}\right\}$$

[2]It can be as high as half the sampling frequency in case of discrete signals.

where K_c is the discrete Fourier transform (DFT) index within the upper-bound K_c^{max} beyond which the value of the magnitude spectrum is below $\overline{V}$. K_c^{max} is the index corresponding to the upper-bound on the cut-off frequency (ω_c^{max}).

5. Compute smoothness.

$$\text{SPARC} \triangleq -\sum_{k=1}^{K_c-1} \sqrt{\left(\frac{1}{K_c-1}\right)^2 + \left(\Delta\hat{V}[k]\right)^2}$$

$$\Delta\hat{V}[k] \triangleq \hat{V}[k] - \hat{V}[k-1], \; k \in \{1, \ldots, K-1\}$$

The Python and Matlab functions for calculating movement smoothness using the SPARC measure can be downloaded from https://github.com/siva82kb/smoothness or from the website of Etienne Burdet.

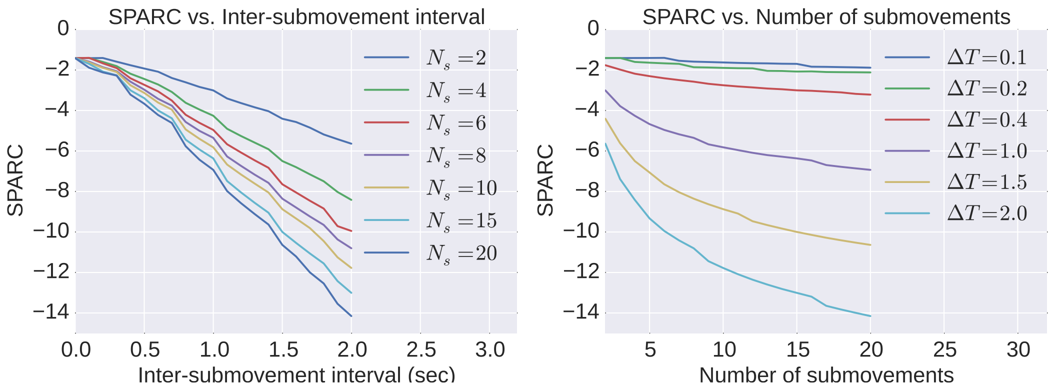

Figure 3: **Results of validation analysis of the SPARC. The left figure shows the plot of the SPARC for the simulated movements as a function the inter-submovement interval, while the right plot shows the same as a function of the number of submovements in the speed profile.**

Validation of SPARC measure

The validation of the SPARC was carried out using simulated data, similar to the one in [1]. The simulated data for the validation of the measure was generated using the following model:

$$v(t) = \sum_{k=1}^{N_s} A_k \, \mathrm{e}^{-25\tau_k^2}, \quad \tau_k = \frac{t - \Delta T_k}{T_k}. \tag{5}$$

The values of the parameters used in the model described above are listed in Table 1 (under the title "Validation analysis"). The smoothness of the simulated movements was numerically computed using the SPARC algorithm. The result of this analysis with simulated data for validating the SPARC is shown in Fig. 3, and it is similar to that of the SAL in [1]. The SPARC was calculated for these different movements using the parameters, $\overline{V} = 0.05$ and $\omega_c^{max} = 20\pi$. The same values were used for the rest of the analysis presented in Appendix B and C.

Appendix B

Reliability of SPARC

The reliability of the SPARC measure was investigated through a simulation analysis similar to the one carried out in [1]. A set of 100 simulated movements X_{ideal} was generated using the model in Eq. 5. The parameters for this model were randomly sampled from uniform distributions over the parameter ranges listed in Table 1 (the parameters ranges are listed under the text "Noise analysis" in this table). Following the generation of X^{ideal}, 25 different realizations of zero-mean white Gaussian noise were added to the 100 movements from X^{ideal}, resulting in a set of 2,500 noisy movements X_{SNR}^{noisy} with a fixed signal-to-noise ratio[3] (SNR); three different noisy movement sets were generated corresponding to SNRs of 100, 10 and 1. The smoothness of all movements from the four data sets X^{ideal}, X_{100}^{noisy}, X_{10}^{noisy}, and X_{1}^{noisy} were estimated using the SPARC and the LDLJ measures.

Table 1: Parameters used for generating the simulated data.

Parameter	Value
**Validation analysis**	
Number of submovements N_s	$\{2, 3, 4, 5, 10, 15, 20\}$
Submovement amplitude A_k	1
Submovement duration T_k	1
Inter-submovement interval $\Delta T_k = \Delta T$[4]	0 to 2
**Noise analysis**	
Number of submovements $N_s \in \mathbb{Z}$	$[1, 10]$
Submovement amplitude $A_k \in \mathbb{R}$	$[0.1, 1.0]$
Submovement duration $T_k \in \mathbb{R}$	$[0.1, 1.0]$
Inter-submovement interval $\Delta T_k \in \mathbb{R}$	$[0.1, 0.5]$

Following the estimation of movement smoothness for the ideal and noisy movement data sets, the effect of noise was quantified by estimating the normalized difference in smoothness of a noisy movement with respect to the range of smoothness values in the ideal data set.

$$\Delta\lambda_{m,n} = 100 \times \frac{\lambda_{m,n}^{noisy} - \lambda_{m}^{ideal}}{\left|\max_m \lambda_m^{ideal} - \min_m \lambda_m^{ideal}\right|}; \;\; m \in \{1, 2, \dots 100\} \text{ and } n \in \{1, 2, \dots 25\} \tag{6}$$

where, $\Delta\lambda_{m,n}$ is the normalized difference in smoothness because of noise, in the nth realization of the mth movement, where m is the movement index and n is the noise index; λ_m^{ideal} is the smoothness estimate (using either SPARC or LDLJ) of the mth ideal movement; and $\lambda_{m,n}^{noisy}$ is the smoothness of the nth noisy realization of the mth ideal movement. The term $\left|\max_m \lambda_m^{ideal} - \min_m \lambda_m^{ideal}\right|$ in the denominator of Eq. 6 is the range of smoothness values in the ideal movement dataset. The normalized difference in smoothness was calculated for all the three noisy datasets with different SNRs.

[3]The variance of the Gaussian noise was scaled according to the signal power to ensure a fixed SNR.

[4]For the validation analysis, the interval between two successive submovements ΔT_k is chosen to be constant (ΔT) for all k.

Fig. 4 shows the plot of the smooth histogram of $\Delta\lambda_{m,n}$s estimated for all the three noisy datasets; negative values for $\Delta\lambda_{m,n}$ indicates lower smoothness for the noisy movement with respect to the corresponding ideal movement. The superior reliability of the SPARC measure with respect to the LDLJ is clear from these three plots. For SNR of 100 and 10, there is relatively no change in the smoothness as estimate by the SPARC measures, while with the LDLJ the smoothness values can change as much as 200% with respect to the actual smoothness value. Even for the case of $SNR = 1$, the smoothness estimated by SPARC changes by a maximum of 25%, while that of LDLJ can change up to 225%.

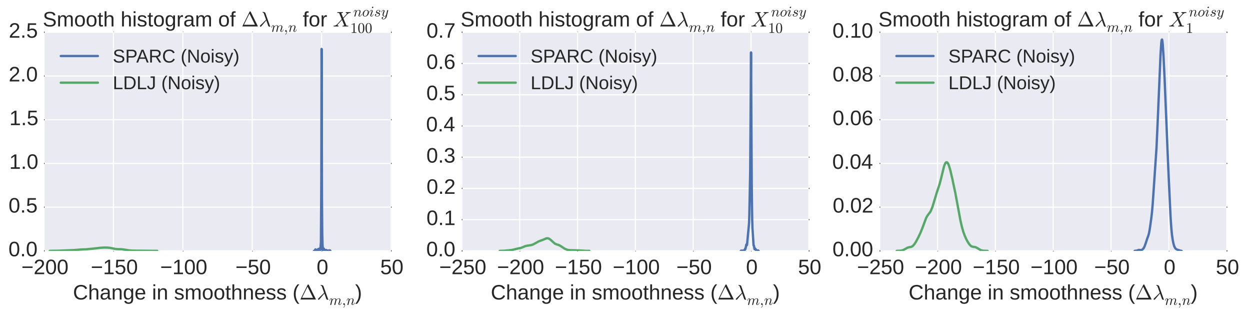

Figure 4: **Plot of the smooth histograms of $\Delta\lambda_{m,n}$ for the noisy dataset of different SNR. The blue trace corresponds to SPARC and the green trace to that of the LDLJ.**

However, the comparison shown in Fig. 4 is an unfair comparison. The SPARC measure uses an inherent lowpass filtering operation through the selection of a cut-off frequency ω_c to estimate smoothness. Thus a fair comparison of the reliabilities of SPARC and LDLJ can be done only after an appropriate lowpass filtering operation on the noisy movement data before estimating smoothness with the LDLJ. In fact, this is the procedure followed in any practical data analysis process.

The choice of filter specification to filter the noisy data can affect the smoothness estimates. A lenient filter (i.e. high cut-off) might not provide significant improvements in the measure's reliability, while stringent filter (i.e. low cut-off) might end up removing use signal components. In addition to the filter cut-off frequency, the choice of filter order might also affect the results. Thus, in order to understand the effects of filter parameters on the reliability of the LDLJ, 6 different lowpass Butterworth filters were used. The 6 filters were the combinations of two filter order ($N = 2$ and $N = 8$), and three cut-off frequencies ($f_c = 10Hz$, $f_c = 15Hz$ and $f_c = 20Hz$). These 6 filters were applied both forward and backward[5] on the three noisy data sets. The smoothness of these filtered datasets was once again estimated using the SPARC and LDLJ measures. The results obtained from the filtered datasets with $SNR = 10$ is shown in Fig. 5. The results for the other two SNRs were similar. It is clear from the plots in Fig. 5 that the use of a lowpass filtering operation significantly improves the reliability of the LDLJ measure, but interestingly its performance is no better than the SPARC measure, applied on the original noisy (un-filtered) data. In fact the filtering appears to have little effect of the SPARC measure, as seen from the blue and green traces in these plots. Moreover, the cut-off frequency appears to have a bigger impact on the effect of noise on the LDLJ, than the order.

[5]This was done to nullify the phase distortion introduced by the forward filter. It must be noted that in effect the overall order of the filter was twice that of the forward filter.

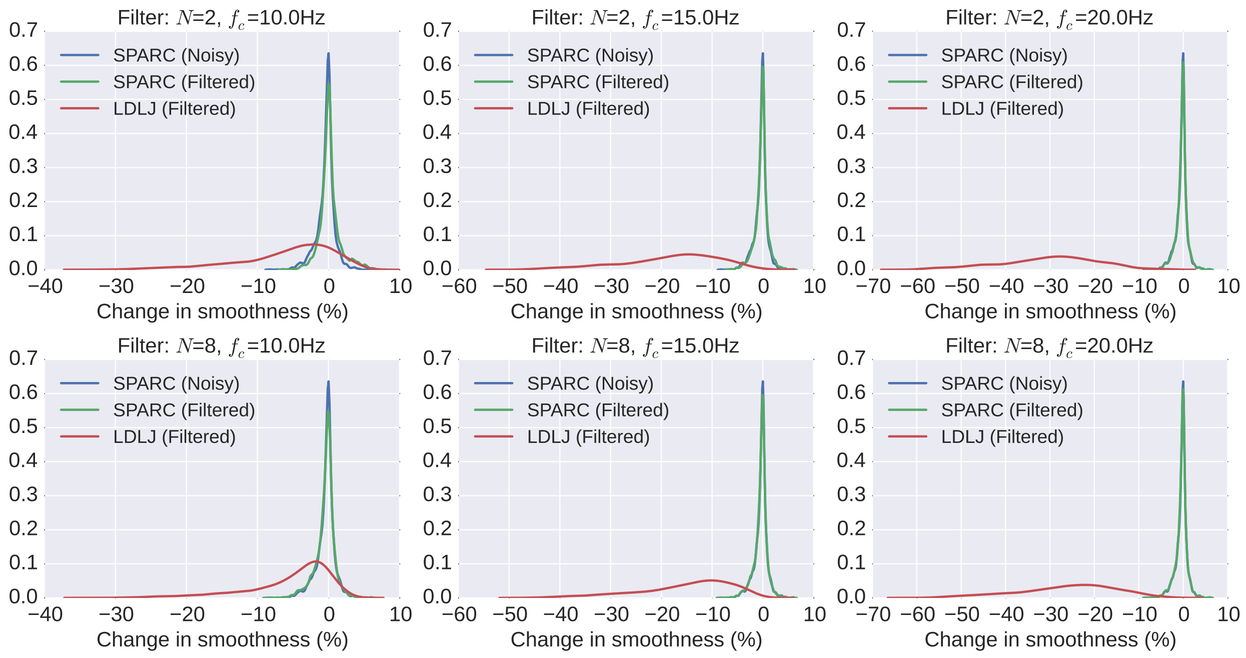

Figure 5: **Plot of the smooth histograms of $\Delta\lambda_{m,n}$ for the filtered dataset with $SNR = 10$. The 6 different plots correspond to the 6 different filters. The blue and green traces correspond to SPARC with the original noisy and filtered data respectively. While the red trace corresponds to the LDLJ applied on the filtered dataset.**

Appendix C

Effect of SNR of movement data on smoothness

To investigate the effect of SNR on the smoothness estimate, a set of 100 simulated movements $X^{fast} = \{x(t)\}$, and movements that are slower versions of X^{fast} were generated $X^{slow} = \{0.5x(0.5t)\}$; the time and amplitude scaling for the slow movements ensure that they correspond to the same task as X^{fast} except for being performed in twice the duration of X^{fast}. Following this, white Gaussian noise of variance 0.01 was added to X^{fast} and X^{slow}, resulting in X^{fast}_{noise} and X^{slow}_{noise} respectively. Similar to the analysis in Appendix B, the noisy movement data was lowpass filtered forward and backward using a 8th order Butterworth filter with a cut off of 10Hz to yield X^{fast}_{filt} and X^{slow}_{filt}. Smoothness of the noisy and filtered movements was estimated using both the SPARC and LDLJ.

The smooth histograms of the smoothness estimates for the fast and slow movements are shown in Fig. 6. The top plot in this figure shows the temporal plot of a movement from X^{fast}_{filt} (blue trace) and the corresponding movement from X^{slow}_{filt} (red trace). The left lower plot shows the smooth histograms of the movement smoothness of X^{fast}_{noise} and X^{slow}_{noise} estimated using the SPARC, while on the right of this plot is the smoothness estimated using LDLJ on X^{fast}_{filt} and X^{slow}_{filt}. Fig. 6 clearly demonstrates that the LDLJ measure underestimates the smoothness of slow movements, compared to that of fast movements, even when the two underlying movements have the same shape for their speed profiles. The main reason for this is the difference in the SNR between the slow and fast movements for a given noise power. On the other hand, the SPARC shows no difference in the smoothness estimates between the fast and slow movements.

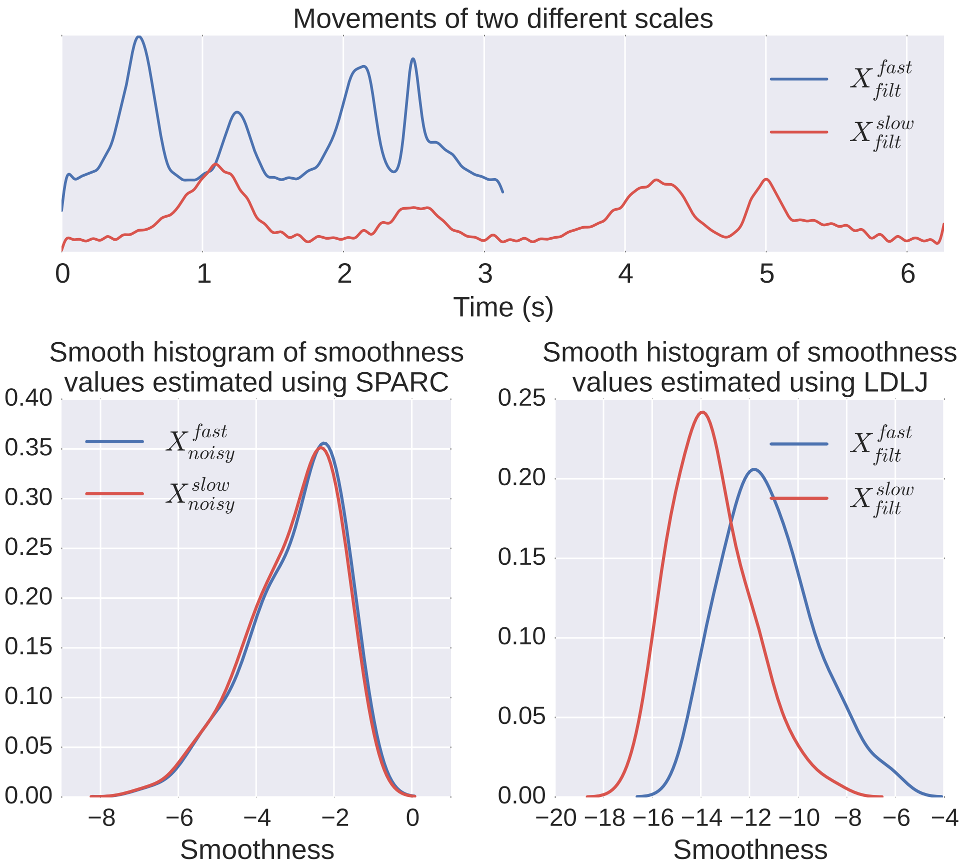

Figure 6: **Plot of the smooth histograms of the different smoothness estimates using SPARC and LDLJ. The SPARC shows the histograms of the noisy fast and slow movements, while the LDLJ shows that of the filtered fast and slow movements. It is clear that even with filtering, the slow movements are esimated to be less smooth than the fast movements by the LDLJ measure.**

References

[1] Balasubramanian, S., Melendez-Calderon, A., Burdet, E.: A Robust and Sensitive Metric for Quantifying Movement Smoothness. IEEE Trans. Biomed. Eng. **59**(8), 2126–2136 (2012)
